# Tuberculosis prevention in children, adolescents, and pregnant and postpartum women in South Africa

**DOI:** 10.4102/safp.v68i1.6198

**Published:** 2026-01-16

**Authors:** Mareike Rabe, Jennifer A. Hughes

**Affiliations:** 1 Family Physician, Private Practice, Vita Oncology, Cape Town, South Africa; 2 Department of Paediatrics and Child Health, Desmond Tutu TB Centre, Faculty of Medicine and Health Sciences, Stellenbosch University, Cape Town, South Africa

**Keywords:** tuberculosis, preventive therapy, children and adolescents, pregnancy, postpartum, primary care

## Abstract

Tuberculosis (TB), particularly drug-resistant TB (DR-TB), remains a major public health concern in South Africa (SA), with children, adolescents, and pregnant and postpartum women (CAPPW) facing heightened risks because of biological and social vulnerabilities. This article highlights the importance of a multipronged prevention framework that combines infection control measures, psychosocial support, education, and nutritional supplementation, alongside pharmacological interventions such as Bacillus Calmette-Guérin (BCG) vaccination and tailored TB preventive therapy (TPT). Drawing on national guidelines and recent academic literature, the article provides an overview of current evidence and recommendations for TPT regimens (including 6H, 3HP, 3RH, 4R, 12H, and 6LFX) and their eligibility, safety considerations, drug interactions, and formulations suitable for CAPPW. By strengthening awareness and streamlining guideline-based prevention efforts, the article equips healthcare workers to make informed, patient-centred decisions to improve treatment outcomes and ultimately reduce TB transmission in high-burden settings.

## Introduction

Tuberculosis (TB) is a disease caused by *Mycobacterium tuberculosis (M.tb)*, an airborne pathogen predominantly transmitted from person to person via inhalation of infected droplet nuclei. Tuberculosis disease can be treated, prevented and controlled using currently available interventions, if implemented comprehensively.

Approximately 212 000 TB cases were notified in South Africa (SA) in 2023, of which 7% were children under the age of 15 years and 37% were among women or adolescent girls of child-bearing potential.^1^ A previous episode of TB disease increases the risk of future TB disease episodes, and approximately 20% of people diagnosed with TB each year have been previously treated for TB.^2^

Drug-resistant (DR)-TB is a disease caused by *M.tb* strains that are resistant to at least rifampicin and/or isoniazid, the two most important first-line drugs used for treating drug-susceptible (DS)-TB. Drug-resistant TB accounts for approximately 3% of SA’s annual burden of new TB cases but demands up to 33% of the total budget for national TB control.^3^ Although a prior diagnosis of any strain of TB is a clear risk factor for future diagnosis of DR-TB, most people diagnosed with DR-TB nationwide have never before received TB treatment, indicating that the majority of incident DR-TB cases are transmitted from person to person within the community (rather than acquired within an individual through suboptimal adherence or inadequate treatment of DS-TB disease).^4^ Furthermore, it was estimated that 13 365 people were diagnosed with DR-TB in SA in 2023 but only 7626 DR-TB cases were notified and started second-line TB treatment^1,5^; this diagnostic-treatment gap contributes considerably to ongoing community transmission of this pathogen.^6^

Transmission of *M.tb* is predominantly airborne in congregate settings, and children, adolescents, and pregnant and postpartum women (CAPPW) with significant TB exposure are at increased risk of TB infection and subsequent progression to TB disease. Other high-risk groups in SA include people living with HIV (PLWHIV), miners, inmates, immunocompromised individuals, those with silicosis and healthcare workers.^7^

Children who are infected and go on to develop TB disease usually do so within 12 months following exposure to someone with pulmonary TB.^8^ Younger age, human immunodeficiency virus (HIV) exposure, HIV infection, and severe malnutrition are risk factors for developing TB disease following significant TB exposure and infection.^9,10,11^ Compared with adults, children are more likely to have successful TB treatment outcomes, but the relatively longer delays in TB diagnosis and appropriate treatment initiation increase their risk of progression to more severe disease. Infants and young children (under 5 years of age) may experience more rapid onset with more severe forms of pulmonary and extrapulmonary TB disease than older children and adolescents (aged 10–19 years) who tend to present with more adult-type (cavitary) pulmonary disease.^9,12^

Adolescents are a unique group of individuals with an increasing desire for autonomy and wider social interactions and are therefore also at risk of TB exposure outside of the household. The risk of progression from TB infection to TB disease increases considerably from the age of 10 years,^8^ and adolescents with significant TB exposure may have distinctly different needs during their developmental transition from children to adults. Clinicians managing adolescents exposed to TB should be mindful of potential challenges associated with their changing perceptions of risk (e.g. experimentation with substance use, engagement in harmful behaviours), willingness to give consent or assent for medical interventions, adherence to treatment or medical advice, and the effects of social isolation and stigma.^13^

Pregnant women infected with TB appear to be at increased risk (compared with non-pregnant women) of progression to TB disease, with an even higher risk during the postpartum period.^14^ Pregnant women with TB disease in SA are more likely to experience unsuccessful TB treatment outcomes compared with non-pregnant women with TB, as well as unfavourable maternal and infant outcomes compared with pregnant women without TB.^15,16^ While the diagnosis and treatment of TB disease during pregnancy and postpartum are clearly beneficial, the optimal management of TB infection during this period remains controversial.^14^

Reducing TB transmission, limiting TB infection, and preventing progression to TB disease among CAPPW are critical to prevent significant morbidity associated with both the disease and the currently available treatment options in these populations. This can be achieved through a combination of multisectoral approaches to promote TB education and awareness, prevent and control *M.tb* transmission, increase antiretroviral therapy (ART) coverage, facilitate routine TB screening, improve diagnosis, prompt initiation of effective TB treatment, and enhance psychosocial support for people and families affected by TB. Provision of TB preventive therapy (TPT) is only one component in the overall approach to achieve control of the TB epidemic in SA. Given the emerging evidence for TPT from international clinical trials and the importance of preventing TB disease in priority populations, this article aims to provide healthcare workers in primary care settings in SA a brief overview of the approach to TB prevention and current TPT recommendations for CAPPW.

Table 1 contains definitions applicable in this article.^7,9^

**TABLE 1:** Definitions.

Term	Definition
**TB infection** (sometimes termed ‘latent TB infection’ in adults)	A state of persistent immune response to stimulation by *Mycobacterium tuberculosis (M.tb)* antigens with no evidence of clinical TB disease. Most infected people have no signs or symptoms of TB but are at risk for TB disease.
**TB disease**	Disease caused by *M.tb* that can be either bacteriologically confirmed or clinically diagnosed (based on history, clinical findings, radiological features, exposure history, and growth trend, where bacteriological tests were either not performed or the results were negative).
**Drug-susceptible (DS)-TB**	*M.tb* strains that are susceptible to the first-line drugs: rifampicin and isoniazid.
**Drug-resistant (DR)-TB**	*M.tb* strains that are resistant to either or both rifampicin and isoniazid.
**TB contact**	Any person (family member and other individual, regardless of age and HIV status) who has had a ‘significant TB exposure’.
**TB index person**	The initially identified person with new or recurrent TB disease in a specific household or other comparable setting in which others may have been exposed. The index person is the centre of the contact investigation but is not necessarily the source of the infection in that setting.
**TB source person**	A person with pulmonary TB disease considered to be the source of TB transmission in a specific setting (not always necessarily the same as the TB index person around whom the contact investigation is initially centred).
**Significant TB exposure**	Exposure (in the 12 months before the TB contact is screened) within the same enclosed space or shared living arrangement for one or more nights, or for frequent or extended daytime periods, to a TB index or source person with pulmonary TB disease during the 3 months before they started TB treatment.

*Source:* Adapted from the South African National Department of Health. National guidelines on the treatment of tuberculosis infection [homepage on the Internet]. 2023 [cited 2025 Jul 06]. Available from: https://knowledgehub.health.gov.za/elibrary/national-guidelines-treatment-tuberculosis-infection and the South African National Department of Health. Management of tuberculosis in children and adolescents [homepage on the Internet]. 2024 [cited 2025 Jul 06]. Available from: https://knowledgehub.health.gov.za/elibrary/management-tuberculosis-children-and-adolescents

Bold text signifies the importance that either or both rifampicin and izoniazid resistance is defined and DR-TB.

TB, tuberculosis; HIV, human immunodeficiency virus.

## General tuberculosis prevention measures

General measures to prevent TB transmission in SA should include: implementation of existing TB infection prevention and control (IPC) policies^17^; community-based activities to increase awareness, education and psychosocial support for people exposed to TB^5,18,19^; nutritional support programmes for families affected by TB^20^; optimised Bacillus Calmette-Guérin (BCG) vaccination coverage^9^; and routine TB contact screening and management.^7^

### Tuberculosis contact screening

**FIGURE 1:**
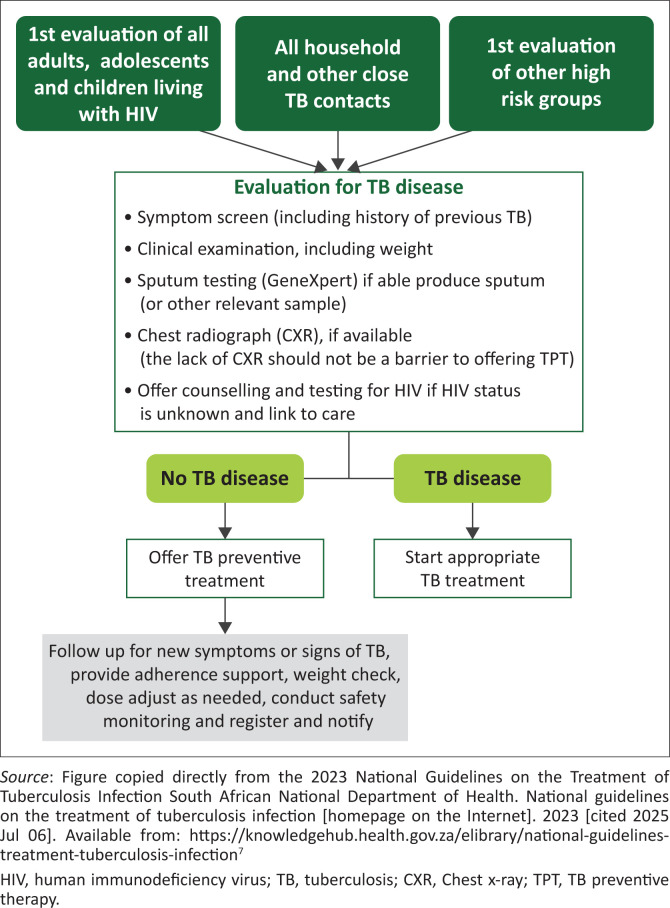
General algorithm for provision of tuberculosis preventive treatment using the test and treat approach.

Once a person has been diagnosed with TB disease, they become the TB index person, and healthcare services should implement routine procedures and processes for the identification and screening of TB contacts as per Figure 1. Some TB contacts will be at higher risk of TB infection and progression to disease than others, and CAPPW should be prioritised for screening. In general, the process for evaluation of TB contacts is the same for establishing TB infection as for diagnosing DS-TB or DR-TB disease.^7,9^

For details of TB screening and evaluation procedures that relate to CAPPW specifically, refer to Section 6 of the 2024 Management of Tuberculosis in Children and Adolescents guideline^9^ and to Section 6 of the 2023 National Guidelines on the Treatment of Tuberculosis Infection.^7^

## Tuberculosis preventive therapy

People of all ages with significant TB exposure, children and adolescents living with HIV, and pregnant and postpartum women are at high risk of developing TB disease and should be screened for TB regularly. The South African National Department of Health (SANDoH) has recommended that individuals from high-risk groups be managed with a ‘test and treat’ approach, whereby TPT is offered to those in whom TB disease has been excluded as per Figure 1.^7,9^

The theory for providing TPT as a preventive measure is that individuals who do not have TB disease but who are infected with viable *M.tb* have a very low bacillary load, which can be eradicated with one or two drugs, thereby reducing the risk of developing TB disease.^21,22^ Therefore, regardless of whether TPT is provided, clinical follow-up of TB contacts, particularly in the 12 months following significant TB exposure, remains critical for early detection and treatment of incident TB disease to reduce morbidity, mortality, and ongoing *M.tb* transmission. Contraindications to TPT include severe liver dysfunction, severe peripheral neuropathy, or previous severe adverse reaction to TPT.^7^

Once a decision has been made to provide TPT for TB infection, the optimal choice of drug or drug combination depends on the effectiveness, availability, side effect profile, cost, drug–drug interactions, drug susceptibility profile of the TB index person, and the risk of resistance amplification.^21^ A recent review article by Chihota et al. provides a comprehensive overview of clinical trial evidence supporting current TPT regimen recommendations, as well as ongoing and future TPT studies relevant to CAPPW.^22^

Table 2 summarises eligibility, composition, duration, and other characteristics of available TPT regimens for CAPPW in SA. For medication dosing guidance in these populations, refer to the relevant guideline documents,^7,9,23,24^ or Section 2 of the Quick Reference Guide.^25^

**TABLE 2:** Overview of available drug-susceptible-TB and drug-resistant-TB preventive therapy regimens in South Africa.

Characteristic	6H	3HP	3HR	4R	12H	6LFX
Drug(s)	Isoniazid	Isoniazid + rifapentine	Isoniazid + rifampicin	Rifampicin	Isoniazid	Levofloxacin
Duration (months)	6	3	3	4	12	6
Frequency	Daily	Weekly	Daily	Daily	Daily	Daily
*M.tb* resistance pattern in TB index/source person	DS-TB; and Rifampicin-mono-resistant TB with confirmed INH susceptibility	DS-TB	DS-TB	DS-TB; and INH-mono-resistant TB	DS-TB; and Rifampicin-mono-resistant TB with confirmed INH susceptibility	Rifampicin-resistant TB with resistance to INH but susceptible to fluoroquinolones
Children and adolescents	All ages; child-friendly (dispersible) formulation available; preferred for children with HIV on LPV/r or NVP.	All ages; child-friendly (dispersible) formulation available. **Not yet recommended for children < 25 kg in South African guidelines.**	All ages; child-friendly (dispersible) formulation available	All ages; no child-friendly formulation available, not generally feasible for children < 25 kg	All ages; child-friendly (dispersible) formulation available; preferred for children and adolescents with HIV initiating DTG or on ART with VL ≥ 50	All ages; child-friendly (dispersible) formulation available
Pregnant women	Conflicting evidence†; not currently recommended in South Africa	Not currently recommended, awaiting clinical trial results	Conflicting evidence†,‡; not currently recommended in South Africa	May be safe, although no safety or efficacy data available for this population‡	Conflicting evidence†; not currently recommended in South Africa	May be safe, although no safety or efficacy data available specifically in this population
Postpartum/breastfeeding women	Safe for use	May be safe, although no safety or efficacy data available for this population	Safe for use	May be safe, although no safety or efficacy data available for this population	Safe for use	May be safe, although no safety or efficacy data available for this population
Interactions with ART§	No restriction	Contraindicated: PIs, NVP, TAF. Use: TDF, EFV, DTG, RAL	Contraindicated: all PIs, NVP. Use with caution: TAF. **Adjust dose: DTG (give 50 mg 12 hourly).** Use: TDF, EFV.	Contraindicated: All PIs, NVP, RAL TAF **Adjust dose: DTG (give 50 mg 12 hourly).** Use: TDF, EFV.	No restriction	No restriction (may interfere with lamivudine clearance)
Side effects and toxicities	Hepatotoxicity (more), peripheral neuropathy, rash, gastrointestinal upset	Flu-like syndrome, hypersensitivity reactions, gastrointestinal upset, orange discolouration of body fluids, rash, hepatotoxicity (less)	Hypersensitivity reactions, hepatotoxicity (less), rash, gastrointestinal upset, hypoprothrombinaemia, orange discolouration of body fluids	Rash, gastrointestinal upset, hepatotoxicity (less), hypoprothrombinaemia, orange discolouration of body fluids	Hepatotoxicity (more), peripheral neuropathy, rash, gastrointestinal upset	Diarrhoea, nausea and bloating, arthralgia, inflamed or torn tendons, muscle pain or weakness, prolonged QTc interval, mood or behaviour changes, insomnia

*Source*: Key characteristics of currently recommended TPT options: Adapted from the WHO Module 1 operational handbook World Health Organization. WHO operational handbook on tuberculosis. Module 1: Prevention – Tuberculosis preventive treatment [homepage on the Internet]. Geneva: WHO; 2024 [cited 2025 Jul 13]. Available from: https://www.who.int/publications/i/item/9789240097773^30^

DS-TB, drug-susceptible tuberculosis; DR-TB, drug-resistant tuberculosis; H/INH, isoniazid; LPV/r, lopinavir-ritonavir; NVP, nevirapine; DTG, dolutegravir; ART, anti-retroviral treatment; PIs, protease inhibitors; TAF, tenofovir alafenamide fumarate; TDF, tenofovir disoproxil fumarate; EFV, efavirenz; RAL, raltegravir.

† , One randomised controlled trial has shown increased risk of poor birth outcomes for mothers taking isoniazid during pregnancy; however, other studies have shown benefits of Isoniazid preventive therapy.^22,31^

‡ , Bleeding attributed to hypoprothrombinaemia has been reported in infants and mothers after use of rifampicin in late pregnancy. Vitamin K is recommended for both mother and infant postpartum if rifampicin is used in the last few weeks of pregnancy.

§ , In women receiving rifamycin-based TPT and oral contraceptives, consider additional barrier contraception methods to prevent pregnancy.

## TB preventive therapy for drug-susceptible tuberculosis

In SA, the current TPT options for DS-TB include: isoniazid and rifapentine given once weekly for 3 months (3HP), daily rifampicin and isoniazid for 3 months (3RH), daily isoniazid for 6 months (6H) or daily isoniazid for 12 months (12H). All people receiving TPT should be offered pyridoxine (vitamin B6) for the duration of their TPT: 25 mg/day if ≥ 5 years and 12.5 mg/day if < 5 years. Lack of pyridoxine supply should not be a reason to withhold or postpone TPT.^7^

Rifampicin has significant drug–drug interactions with many ART drugs, and dolutegravir (DTG) dosing should be boosted if given with rifampicin-based TPT (see Table 2). Isoniazid and rifapentine do not interact significantly with DTG and are therefore preferred in PLWHIV. The PLWHIV may be offered 3HP if they are already virally suppressed (viral load < 50 copies/mL) on a DTG-containing regimen. However, those recently *initiating* DTG-based ART regimens or are virally unsuppressed should rather be offered 12H instead of 3HP. The reason is that rifapentine and DTG are metabolised by the same liver enzymes (CYP3A and UGT1A1). The DOLPHIN trial^26^ showed that while DTG levels dropped by ~60% during co-administration with 3HP, most participants maintained viral suppression if they were already virally suppressed on DTG-based ART. New evidence to support simultaneous initiation of DTG-based ART and 3HP is emerging,^27^ and guidelines may be updated to reflect this in future.

### Children and adolescents

Unfortunately, 3HP is not yet available in SA for children weighing < 25 kg. Once the DTG levels from the DOLPHIN-TOO,^27^ DOLPHIN-kids,^28^ and TBTC Study 35^29^ trials are available, 3HP will likely be the preferred option in all PLWHIV. Dispersible, scored and child-friendly fixed-dose combinations (FDCs) of 3RH are routinely available and should be used for HIV-uninfected children weighing < 25 kg. For children living with HIV and *initiating* a DTG-containing ART regimen, 6H is currently the preferred regimen in those weighing < 25 kg.

For children and adolescents weighing ≥ 25 kg, multiple TPT options are available, but 3HP is generally preferred, unless specifically contraindicated. For HIV-uninfected children weighing ≥ 25 kg, 3RH and 6H are alternative options if 3HP is unavailable. Children and adolescents living with HIV may be offered 3HP if they are already virally suppressed (viral load < 50 copies/mL) on a DTG-containing regimen. However, those recently *initiating* DTG-based ART regimens or are virally unsuppressed should rather be offered 12H instead of 3HP, at least until the results of the DOLPHIN-kids trial^28^ and TBTC Study 35 trial^29^ are available.

### Pregnant and postpartum women

The evidence base for TPT during pregnancy remains weak, with conflicting evidence regarding the optimal timing and safety of isoniazid-based TPT in pregnant and postpartum women.^22,30^ The 2023 National Guidelines on the Treatment of Tuberculosis Infection recommended 12H for pregnant women living with HIV and 3RH or 6H for TB-exposed pregnant women without HIV.^7^ However, in January 2025, the SANDoH issued a circular with revised guidance that initiation of isoniazid-based preventive therapy (IPT) should be deferred in all pregnant women, regardless of HIV status, until after delivery, citing an increased risk of adverse pregnancy outcomes after IPT exposure in pregnancy.^32^ This risk was considered to outweigh the benefit of IPT in pregnant women living with HIV with high CD4 counts (> 350 cells/mm^3^). The circular was updated in December 2025 to recommend IPT in pregnancy for women living with HIV. It recommends deferring IPT until after delivery if CD4 counts are above 200 cells/mm^3^, but initiating 12 months of IPT during pregnancy if CD4 counts are 200 cells/mm^3^ or lower, after excluding active tuberculosis, with routine TB screening at each antenatal visit.^33^ Globally, the World Health Organization (WHO) recommends the use of isoniazid and/or rifampicin-based TPT in pregnancy, regardless of the degree of immunosuppression.^34^ While 3HP is not currently recommended for TPT in pregnant or postpartum women because of limited supporting evidence, the results of an ongoing clinical trial, DOLPHIN-Moms, are expected to inform future guidance for women living with HIV.^35^

## TB preventive therapy for drug-resistant tuberculosis

The 2023 National Guidelines on the Treatment of Tuberculosis Infection do not include recommendations for preventive therapy regimens for DR-TB.^7^ Although supporting evidence is scarce, the 2019 Clinical Management of Rifampicin-Resistant Tuberculosis guideline does suggest possible options for treatment of DR-TB infection, depending on the resistance pattern of the *M.tb* strain.^23^

### Children and adolescents

Recently published evidence from the TB-CHAMP and VQUIN trials supports the use of daily levofloxacin for 6 months (6Lfx) in children aged ≤ 5 years living with or without HIV, and older children and adolescents aged < 18 years living with HIV, following significant exposure to fluoroquinolone-susceptible DR-TB strains.^36,37,38^ In SA, children and adolescents with significant DR-TB exposure may be eligible to receive the following TPT options: 6Lfx (for rifampicin- and isoniazid-resistant fluoroquinolone-susceptible TB), daily isoniazid for 6 months (6H, for rifampicin-mono-resistant TB with confirmed phenotypic susceptibility to isoniazid), or daily rifampicin for 4 months (4R, for isoniazid-mono-resistant TB).^23,25^

### Pregnant and postpartum women

The national circular with revised guidance for TPT in pregnancy does not apply to pregnant women exposed to DR-TB that includes isoniazid resistance^31^; however, there is also no definitive national or international recommendation specifically for TPT in pregnant and postpartum women with significant DR-TB exposure.^34^ The 2019 Clinical Management of Rifampicin-Resistant Tuberculosis guideline^23^ suggests possible TPT options (including levofloxacin, high-dose isoniazid and/or delamanid) for DR-TB household contacts, which would arguably include pregnant and postpartum women; however, there are no clinical trial data to either support or contradict these options in this population. Therefore, clinicians and pregnant women wishing to consider TPT following significant DR-TB exposure should consider the risks and benefits of available options and consult an expert for advice if needed.

## Additional considerations

For TPT and ART interactions, refer to Table 2. Use interaction checker tools, such as EMGuidance or the SA HIV/TB Hotline application to check for other significant drug–drug interactions with TPT.

Table 2 summarises common side effects and toxicities. Most side effects are mild and can be managed symptomatically. All adverse events should be reported.

People on TPT should be reviewed monthly. For guidance around TPT treatment interruption, refer to Section 6 of the 2023 National Guidelines on the Treatment of Tuberculosis Infection.^7^

## Conclusion

Preventing TB among CAPPW requires a co-ordinated and individualised approach. Through timely screening, IPC, psychosocial support, and targeted TPT, primary care providers play a critical role in mitigating TB-related morbidity and community transmission. As evidence evolves, particularly regarding TPT use in CAPPW, ongoing education and guideline adherence remain vital. Empowering clinicians with practical tools and clinical insight will enable safer, more effective TB prevention in SA’s most vulnerable populations.
